# Interactive deep learning for myocardial scar segmentation using cardiovascular magnetic resonance

**DOI:** 10.1016/j.jocmr.2026.102720

**Published:** 2026-03-20

**Authors:** Aida Moafi, Danial Moafi, Simran Shergill, Evgeny M. Mirkes, David Adlam, Nilesh J. Samani, Gerry P. McCann, Mostafa Mehdipour Ghazi, J. Ranjit Arnold

**Affiliations:** a Department of Cardiovascular Sciences, University of Leicester, the National Institute for Health and Care Research Leicester Biomedical Research Centre and British Heart Foundation Centre of Research Excellence, Glenfield Hospital, Leicester, UK; b Department of Information Engineering and Mathematics, University of Siena, Siena, Italy; c Department of Mathematics, University of Leicester, Leicester, UK; d Pioneer Centre for AI, Department of Computer Science, University of Copenhagen, Copenhagen, Denmark; e Centre for Digital Health and Precision Medicine, University of Leicester, Leicester, UK

**Keywords:** Myocardial infarction, Scar segmentation, Deep learning, Foundation model, Clinical artificial intelligence, Human-in-the-loop

## Abstract

**Background:**

Following myocardial infarction, late gadolinium enhancement (LGE) assessed by cardiovascular magnetic resonance (CMR) provides a reliable metric for risk stratification and therapeutic planning. However, conventional segmentation methods are time-consuming and labor-intensive, with high inter-observer variability and inconsistent performance in routine clinical practice. This study sought to develop an interactive deep learning system for scar segmentation and quantification.

**Methods:**

The framework was developed and evaluated using LGE-CMR images from 348 patients with chronic myocardial infarction (244 training, 51 validation, and 53 test). The model incorporates prompt-guided segmentation and leverages a vision foundation model adapted for medical imaging, integrated into a clinician-facing interface for real-time interaction, and automated quantification. Training used a composite loss function combining Dice overlap, voxel-wise cross-entropy, and Kullback–Leibler divergence against soft labels to address annotation uncertainty. Performance was evaluated on a held-out test set using expert manual annotations as the reference standard, with assessment of segmentation accuracy, repeatability, and agreement with the conventional full-width at half-maximum method (FWHM).

**Results:**

The framework achieved expert-level segmentation performance on the test set (Dice similarity coefficient = 0.74 ± 0.10; Hausdorff distance = 5.87 ± 6.79 mm) with a median scar mass error of 1.28 g (interquartile range [IQR] 0.74–2.34), corresponding to 1.4% (IQR 0.81–2.47) of left ventricular mass. Repeatability analysis (n = 41) demonstrated excellent agreement, with both inter- and intra-observer concordance correlation coefficients of 0.999 (compared with 0.737 and 0.952, respectively, for the conventional FWHM). Segmentation time was substantially reduced when using the interactive tool compared with the conventional workflow, averaging 65 ± 34 s per patient. Performance and repeatability remained high across the test set with differing levels of image quality.

**Conclusion:**

The proposed framework for scar segmentation with a *human-in-the-loop* design enables fast, accurate, and highly reproducible myocardial scar quantification from LGE-CMR. This may provide more consistent performance in routine clinical workflows.

## Graphical abstract

**Figure:**
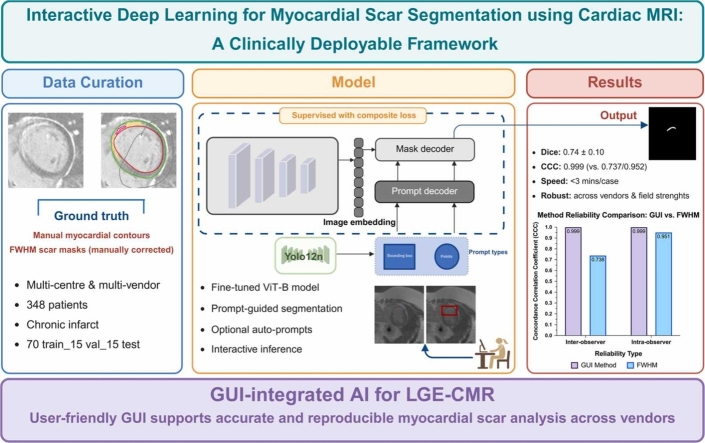

## 1 Introduction

Coronary artery disease remains the leading cause of death and disability worldwide, imposing a substantial health and economic burden globally [1], [2]. In the United Kingdom alone, 1.4 million people have survived a myocardial infarction (MI) [3], the extent of which is a powerful prognostic indicator: each 5% increase in scar size is associated with a 19% higher risk of mortality [4]. Furthermore, scar burden underlies a number of outcomes post-MI, including progression to heart failure, the likelihood of ventricular arrhythmia, and response to revascularization [5], [6], [7], [8]. Hence, there is a pressing need for clinically applicable tools for accurate and reproducible scar quantification [9].

Late gadolinium enhancement cardiovascular magnetic resonance (LGE-CMR) is the gold standard for the non-invasive detection and quantification of myocardial scar [10]. However, conventional methods for scar quantification rely on manual segmentation of images, which is time-consuming, operator-dependent, and subject to significant inter- and intra-observer variability. Semi-automated thresholding techniques such as signal threshold versus reference mean and the full-width at half-maximum method (FWHM) were introduced to reduce manual workload and standardize quantification [11], [12]. While these methods represented an important step forward, they still depend on manual myocardial delineation and user-defined parameters, resulting in inconsistent performance across varying image quality, scanner vendors, and institutional protocols [13], [14], [15], [16], [17], [18].

To overcome these limitations, deep learning-based approaches have been developed, which directly predict scar regions from LGE-CMR images [19], [20], [21]. However, most existing models are trained on small, homogeneous datasets, limiting their generalizability. Even in benchmarking challenges such as the automatic Evaluation of Myocardial Infarction from Delayed-Enhancement Cardiac MRI (EMIDEC), leading models struggled with subtle or diffuse enhancement patterns, underscoring the need for more robust solutions [22].

To address these challenges, we developed an interactive artificial intelligence (AI) system for myocardial scar segmentation. Our prior work [23] detailed architecture selection and comparison with alternative segmentation models; the present study extends this with a human-in-the-loop framework. This work combines automated prediction with a lightweight graphical user interface (GUI) for real-time refinement, and evaluates different prompting strategies and repeatability.

## 2 Methods

### 2.1 Study design and population

**Fig. 1:**
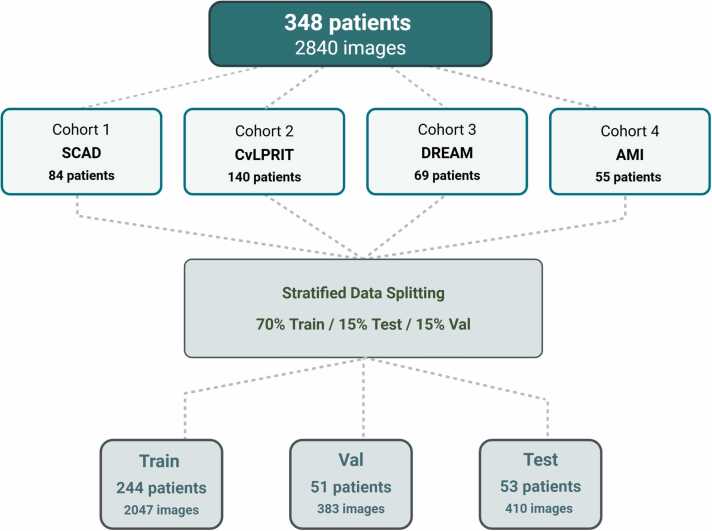
Overview of dataset composition and partitioning. Four cohorts contributed 348 patients (2840 images). Stratified sampling produced training (n = 244; 2047 images), validation (n = 51; 383 images), and test (n = 53; 410 images) sets. Segmentation accuracy (DSC, HD) and scar mass error were evaluated in the full test set (n=53). Repeatability analysis (inter- and intra-rater agreement) was performed in a subset (n=41) based on prospective sample size calculation. *DSC* Dice similarity coefficient, *HD* Hausdorff distance, *CvLPRIT* Complete versus Lesion-only Primary Percutaneous Coronary Intervention trial, *DREAM* Daily Remote Conditioning in Acute Myocardial Infarction trial, *SCAD* Spontaneous Coronary Artery Dissection registry, *AMI* acute myocardial infarction

This study retrospectively analyzed LGE-CMR images acquired from four independent patient cohorts with a history of ST-segment elevation MI during the chronic post-infarction phase (between 3 and 12 months). A total of 348 subjects were included in the analysis, recruited between 2010 and 2020 across multiple institutions and scanner platforms (Fig. 1, Table 1). Data from four cohorts were derived from previously published prospective studies SCAD [Spontaneous Coronary Artery Dissection (UK National Registry)], CvLPRIT [Complete versus Lesion-only Primary Percutaneous Coronary Intervention (ISRCTN70913605)], DREAM [Daily Remote Conditioning in Acute Myocardial Infarction (NCT0166461)], and AMI [Acute Myocardial Infarction (Derbyshire Research Ethics Committee, 09/H0401/21)] [24], [25], [26], [27].

**Fig. 2:**
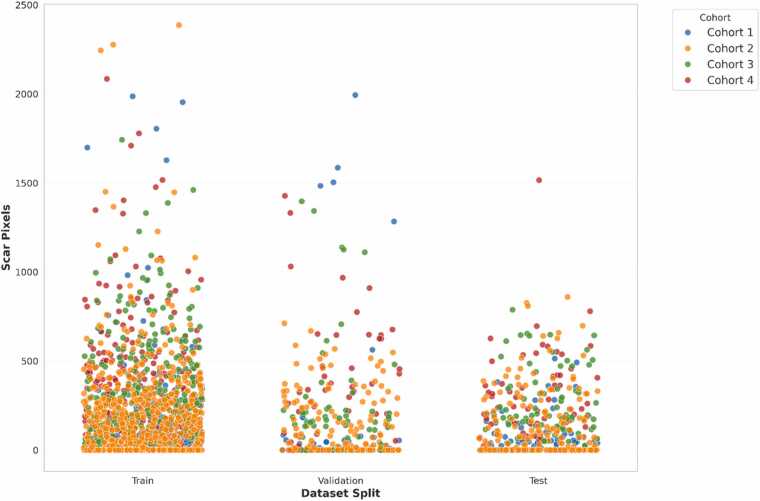
Scar pixel distribution by split and cohort. Distribution of scar pixel counts across training, validation, and test sets, stratified by cohort. Each dot represents a single image. No statistically significant differences were observed across the splits (Kruskal–Wallis H = 0.608, *p*-value = 0.738), confirming the test set is representative of the overall dataset

Patients from each cohort were selected based on three prespecified criteria: (i) short-axis LGE-CMR providing complete coverage of the left ventricle, (ii) visual image quality sufficient for manual annotation (i.e., absence of major artifacts or poor contrast-to-noise ratio, as judged by an experienced reader; Supplementary Fig. S1), and (iii) availability of essential clinical data (e.g., infarct timing, age, and sex). Patients were partitioned at the subject level into training (70%; 244/348), validation (15%; 51/348), and internal test (15%; 53/348) subsets using stratified sampling based on total scar burden. This ensured balanced representation of infarct size across sets and mitigated potential bias due to variability in myocardial damage (Fig. 2). This multicohort design allowed for robust evaluation of model performance and improved generalizability across diverse clinical settings. We have previously published additional details on the broader dataset and its previous use in a fully automated segmentation framework [23].

All studies were conducted in accordance with the Declaration of Helsinki and approved by local research ethics committees. Written informed consent was obtained before enrollment, and all data were anonymized before transfer and analysis.

### 2.2 Image acquisition

LGE-CMR scans were acquired using 1.5T or 3T magnetic resonance systems (Siemens Skyra or Avanto, Siemens Healthineers, Erlangen, Germany; Philips Intera, Best, the Netherlands), with in-plane resolutions ranging from 1.17 to 1.96 mm and slice thicknesses of 6 to 10 mm, and a slice gap of 10 mm, depending on institutional protocols (Supplementary Table S1). Full left ventricular coverage was achieved using contiguous short-axis stacks (typically 10–12 slices). Scans were electrocardiography-gated and acquired during an end-expiratory breath-hold using standard inversion recovery sequences, performed 10–20 min after intravenous administration of 0.1–0.2 mmol/kg of a gadolinium-based contrast agent (depending on study protocol). Phase-sensitive inversion recovery (PSIR) reconstructions were analyzed when available, which provided improved scar–myocardium contrast and reduced sensitivity to inversion time selection. In earlier datasets lacking PSIR sequences, magnitude images were analyzed.

### 2.3 Ground truth annotation

Manual ground truth annotations were performed by a single experienced reader using certified software (cvi42, Circle Cardiovascular Imaging, Calgary, Alberta, Canada (version 5.6)). Endocardial and epicardial contours were manually drawn for each short-axis LGE-CMR slice to define myocardial borders. Scar segmentation was performed using the FWHM method, with a region of interest placed in the area of peak enhancement within the infarct core, and voxels exceeding 50% of the peak intensity were automatically labeled as scar [12]. To refine these masks, manual exclusion zones were applied in cases where enhanced pixels, whether due to noise, artifact, or partial volume effects, were incorrectly included by the thresholding. The final corrected scar masks were used as the ground truth for model training and validation.

### 2.4 Data pre-processing

All LGE-CMR images were standardized to a uniform in-plane resolution of 256 × 256 pixels using bicubic interpolation. Spatial scaling was computed from Digital Imaging and Communications in Medicine (DICOM) header metadata to preserve anatomical aspect ratios across different scanner vendors and institutions. To mitigate variability in signal intensity across acquisitions, per-slice intensity normalization was performed. Pixel values were clipped at the 0.1st and 99.9th percentiles, followed by z-score standardization. This normalization strategy was applied independently to each slice to mitigate local inhomogeneities while preserving contrast between scar and healthy myocardium.

### 2.5 Data augmentation

To enhance robustness to anatomical variability and scanner-dependent artifacts, extensive data augmentation was applied during training. These augmentations targeted both spatial and intensity domains and were implemented using a modular pipeline built on the TorchIO framework [28], which is specifically designed for pre-processing and augmentation of medical imaging data. Spatial augmentations included random in-plane rotations (±15^◦^), isotropic scaling (±10%), affine shearing, and random elastic deformations were applied using a 7 × 7 grid of control points with up to 7.5 mm displacement, enabling smooth non-rigid warping of each slice to simulate anatomical variability. All spatial transformations were applied identically to both the input images and their corresponding ground truth masks to maintain spatial alignment.

**Fig. 3:**
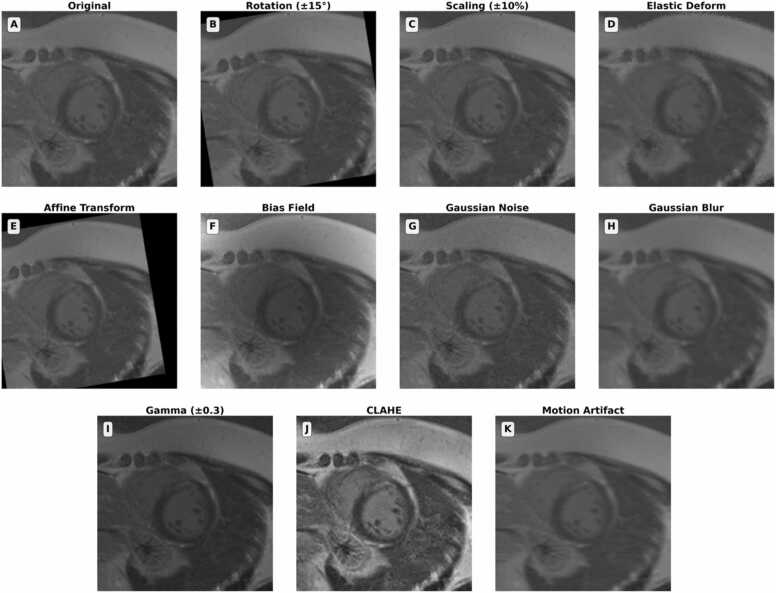
Data augmentation pipeline for SAM fine-tuning. Illustration of the data augmentation pipeline used for fine-tuning SAM on cardiac MRI. (A) Original short-axis slice with visible myocardial scar. (B–E) Geometric augmentations: rotation (±15^◦^), scaling (±15%), elastic deformation (±3 pixels), and affine transformations to simulate anatomical and acquisition variability. (F–H) Intensity augmentations: bias field distortion, Gaussian noise, and blurring to replicate scanner artifacts and motion-related degradation. (I–J) Contrast augmentations: gamma correction (γ = 1.3) and CLAHE to simulate differences in scanner calibration and enhance local tissue contrast. (K) Simulated motion artifacts mimicking cardiac and respiratory motion. All transformations were applied probabilistically during training to increase model robustness while preserving anatomical structure. *SAM* segment anything model, *MRI* magnetic resonance imaging, *CLAHE* contrast-limited adaptive histogram equalization

To model scanner-induced artifacts and acquisition imperfections, intensity-level augmentations included bias field distortions (implemented as third-order polynomial fields with coefficient magnitudes up to 0.5), Gaussian noise addition (zero-mean noise with standard deviation *σ* ∈ [0, 0*.*1]), and mild Gaussian blurring to simulate loss of sharpness. Gamma modulation (log-space gamma range ± 0.3) was used to mimic nonlinear contrast scaling, and contrast-limited adaptive histogram equalization (CLAHE) [29] enhanced local myocardial texture while preserving global intensity structure. All augmentations were applied probabilistically on a per-sample basis during training (CLAHE to all samples; flips and gamma correction at 50%; all other augmentations at 30%) and jointly to image–mask pairs to preserve spatial correspondence. This augmentation strategy was designed to reflect plausible clinical variability encountered in LGE-CMR across sites, scanners, and patient populations (Fig. 3).

### 2.6 Model setup and zero-shot evaluation

We evaluated three backbones of the segment anything model (SAM) [30] for myocardial scar segmentation on LGE-CMR: three standard vision transformer variants (ViT-B, ViT-L, and ViT-H) and MedSAM [31], a version pretrained on medical imaging data. All models were assessed in a zero-shot setting using publicly available weights. Bounding box prompts were automatically generated by computing the minimal enclosing rectangle around each ground truth mask, with a 2-pixel margin added and applied uniformly across all test subsets. Segmentation accuracy was evaluated using the Dice similarity coefficient (DSC). Architecture selection was informed by prior benchmarking against state-of-the-art segmentation architectures (nnU-Net, DeepLabv3+), which were trained from scratch on the same dataset without pretrained weights [23].

### 2.7 Loss function design

To supervise training, we implemented a composite loss function that balances region-level accuracy, voxel-wise classification, and uncertainty regularization. Specifically, the total loss is defined as:

$$L_{\text{total}}=\lambda_{\text{Dice}}\cdot L_{\text{Dice}}+\lambda_{\text{BCE}}\cdot L_{\text{BCE}}+\lambda_{\text{KL}}\cdot L_{\text{KL}}$$

where L_Dice_ promotes spatial overlap between the predicted and reference masks, L_BCE_ penalizes voxel-wise misclassifications, and L_KL_ (Kullback-Leibler; KL) encourages uncertainty-aware learning by quantifying the divergence between predicted and soft-label probability distributions, reducing overconfidence at uncertain scar–myocardium boundaries. The individual components are defined as follows:

$$L_{\text{Dice}}=1-\frac{2\sum_{i}p_{i}g_{i}+\epsilon }{\sum_{i}p_{i}+\sum_{i}g_{i}+\epsilon }$$

$$L\text{BCE}=-\frac{1}{N}\sum_{i=1}^{N}\left[g_{i}\log\left(p_{i}\right)+\left(1-g_{i}\right)\log\left(1-p_{i}\right)\right]$$

$$L_{\text{KL}}=\sum_{i}s_{i}\log\left(\frac{s_{i}}{p_{i}+\epsilon }\right),$$

where *p*_*i*_ denotes the predicted probability at voxel *i*, *g*_*i*_ is the binary ground truth, and *s*_*i*_ is the corresponding soft label. *ϵ* is a small constant for numerical stability. To generate soft labels *s*_*i*_, we applied a Gaussian filter with standard deviation *σ* to the binary ground truth masks. This smoothing reduces sharp intensity transitions at the scar–myocardium boundary and captures annotation uncertainty. A grid search was performed to determine the optimal *σ* value based on validation performance. We found that *σ* = 2 yielded the best DSC score and used this value in all subsequent experiments. Final loss weights (*λ* values) were optimized via Bayesian search on a held-out validation set. The selected configuration was normalized to ensure stability. This balanced formulation was found to maximize generalization while preserving structural consistency in the segmentation outputs.

### 2.8 Prompting strategy

To determine the most effective prompting configuration for interactive segmentation, we fine-tuned the model using three variations: (1) bounding box only, (2) positive points only, and (3) a combined box-and-point strategy. Performance was compared across these setups using validation data, and the combined configuration, where both box and points were derived from the bounding box, consistently yielded the best results. Additionally, to reduce model sensitivity to prompt variability and improve robustness to human interaction, we implemented prompt augmentation during training. Bounding boxes were randomly shifted (±10 pixels) and expanded (up to 20%) to simulate inconsistent box placement. For point-based prompting, 2–10 positive points were sampled within each scar region. This approach ensured stable performance across a range of input styles and interaction variability.

### 2.9 Fine-tuning approach

Fine-tuning was conducted using the ViT-B variant of the SAM, selected for its superior performance in the zero-shot evaluation. The training pipeline updated both the image encoder and mask decoder components, while the prompt encoder was kept frozen to retain general-purpose representations learned during pretraining. Model optimization employed the AdamW optimizer with an initial learning rate of 1 × 10^−4^ and weight decay of 1 × 10^−2^. A stepwise decay schedule reduced the learning rate by a factor of 0.5 every 10 epochs. The hyperparameter tuning and model checkpoints were conducted using the Weights & Biases platform [32].

Input data comprised pre-processed LGE-CMR slices with corresponding binary scar masks and structured prompts. All inputs adhered to the standardized pipeline for spatial normalization and resolution adjustment. Training proceeded for a maximum of 100 epochs, with early stopping triggered after 20 consecutive epochs without improvement in validation DSC score. Experiments were executed on a high-performance computing cluster with 12 central processing unit (CPU) cores, 128 GB system memory, and one NVIDIA A100 GPU (40 GB VRAM, NVIDIA, Santa Clara, California).

### 2.10 Interactive interface design

To support user-guided myocardial scar segmentation in both clinical and research workflows, we developed a lightweight GUI in Python using the Tkinter framework. The tool enables real-time model interaction, intuitive input manipulation, and quantitative assessment in a unified environment. The GUI supports multi-format image loading, including DICOM, Neuroimaging Informatics Technology Initiative, and Portable Network Graphics, and performs segmentation using bounding box and foreground point prompts. Initial bounding boxes are automatically suggested via a You Only Look Once (YOLO12n) detector, streamlining the interaction pipeline. Users may further refine predictions by interactively adjusting the bounding box or adding foreground points. In brief, the detector model automatically suggests a rectangle around the scar, which the user can adjust or redraw; clicks (point prompts) help direct the segmentation model's attention (Supplementary Fig. S2). To correct segmentation artifacts and accommodate user preferences, the interface incorporates morphological operations for mask shrinkage and expansion (3 × 3 elliptical kernel, single iteration), which allow direct geometric modification of predicted contours. These tools emulate realistic user correction behavior, enabling fine-tuning of scar boundaries post-segmentation. For quantitative analysis, the GUI parses spatial metadata from DICOM headers to extract in-plane resolution and slice thickness. This information is used to compute the myocardial scar mass on a per-slice basis and cumulatively across the volume. Voxel-wise integration is performed assuming a myocardial tissue density of 1.05 g/cm^3^, and the interface logs the scar mass for each slice and the total scar burden. This log is accessible within the session and exportable as a structured file for record-keeping or further analysis.

Additional utilities include gamma correction for dynamic contrast adjustment, batch loading for multi-slice navigation, keyboard-based input control, and export of segmentation masks and annotated prompts. All computations are executed locally to maintain full compliance with data privacy regulations and to enable seamless deployment in offline clinical environments (see Supplementary Video S1 as a demonstration video of the interface, including prompt editing, real-time inference, and scar mass computation).

Supplementary material related to this article can be found online at doi:10.1016/j.jocmr.2026.102720.

The following is the Supplementary material related to this article Video S1..

Video S1

### 2.11 Repeatability analysis

To assess clinical applicability and repeatability, the fine-tuned model was evaluated on 41 independent chronic post-ST-segment elevation MI patients. Sample size was calculated using Bonett’s method for the intraclass correlation coefficient (ICC) of the average of two raters. Assuming an expected ICC of 0.90 and a desired 95% confidence interval width of 0.10, a minimum of 38 participants was required [33]. We included 41 patients in the repeatability cohort, which exceeds this requirement and ensures the targeted precision of the ICC estimate. Each case was segmented by two expert readers using both the conventional FWHM method and the GUI-based method. Expert A performed two independent segmentation rounds (≥7 days apart) for both the FWHM- and GUI-based methods to assess intra-observer repeatability. Expert B completed a single round of segmentation with each method, enabling inter-observer repeatability analysis. All experts were blinded to the reference annotations and each other’s results. GUI-based segmentation involved prompt-driven interaction using an automatically generated bounding box, with optional refinement and manually added foreground points. Both experts received a brief GUI orientation (∼10 min) and were instructed to adjust bounding boxes to encompass the full scar and place point prompts within enhanced regions.

### 2.12 Statistical analysis

Segmentation performance was evaluated using voxel-wise DSC and Hausdorff distance (HD), computed on a per-slice basis across the test set. These metrics were used to quantify spatial overlap and boundary accuracy between predicted and expert-annotated masks, providing a comprehensive assessment of model precision at the image level. One-way analysis of variance (ANOVA) was used to compare segmentation performance across infarct territories. To assess clinical relevance, scar mass error was calculated as the absolute difference between model-derived and ground truth scar mass, expressed in grams and as a percentage of left ventricular myocardial mass. To assess repeatability, scar mass estimates were compared between observers using Pearson’s correlation coefficient (r), Lin’s concordance correlation coefficient (CCC), ICC, Bland–Altman analysis, and two-tailed paired t-tests to evaluate for systematic bias. Data were presented as mean ± standard deviation (SD) or median (interquartile range [IQR]) where appropriate. These comparisons were performed separately for both GUI- and FWHM-based annotations. A *p*-value <0.05 was considered statistically significant. Statistical analyses were conducted in Python version 3.11 using the SciPy, Pingouin, and Statsmodels libraries.

## 3 Results

### 3.1 Zero-shot performance with pretrained foundation models

**Fig. 4:**
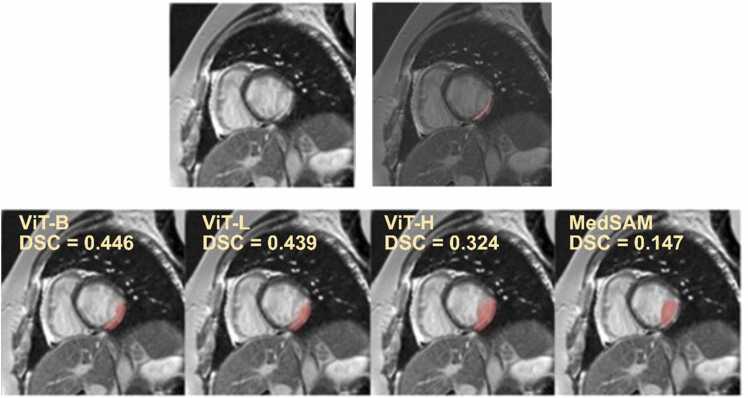
Zero-shot segmentation outputs from multiple SAM variants. Zero-shot segmentation results from four SAM variants on a representative LGE-CMR slice. Red overlays indicate predicted scar regions. *SAM* segment anything model, *LGE* late gadolinium enhancement, *CMR* cardiovascular magnetic resonance, *DSC* Dice similarity coefficient, *MedSAM* medical segment anything model, *ViT* vision transformer

We first evaluated the zero-shot segmentation performance of four SAM variants without domain-specific fine-tuning. All models demonstrated limited segmentation accuracy on LGE-CMR scar images, with DSC scores ranging from 0*.*33 ± 0*.*23 (MedSAM) to 0*.*40 ± 0*.*22 (ViT-B) (Table 2). Qualitative examples of zero-shot predictions are illustrated in Fig. 4, highlighting the relative superiority of ViT-B and ViT-L in capturing scar regions. Among all variants, ViT-B achieved the highest DSC score while offering a favorable trade-off between segmentation accuracy (DSC = 0*.*40 ± 0*.*22), inference speed (37.54 s), and model complexity (86 M parameters). Given this balance of performance and efficiency, ViT-B was selected as the base architecture for subsequent fine-tuning and evaluation.

**Table 1:** Demographic characteristics of the included cohorts.

Cohort	Patients	Female, n (%)	Age (y)
SCAD (cohort 1)	84	55 (65)	45.41±21.20
CvLPRIT (cohort 2)	140	24 (17)	67.35±11.71
DREAM (cohort 3)	69	17 (25)	59.44±9.85
AMI (cohort 4)	55	6 (11)	59.51±10.71

*AMI* acute myocardial infarction, *CvLPRIT* Complete versus Lesion-only Primary Percutaneous Coronary Intervention trial, *DREAM* Daily Remote Conditioning in Acute Myocardial Infarction trial, *SCAD* Spontaneous Coronary Artery Dissection registry

Data are presented as n (%) or mean ± standard deviation.

**Table 2:** Zero-shot segmentation performance of SAM variants.

Model	DSC	Inference time (s)	Inference time/slice (s)	Parameters (million)
SAM ViT-H	0*.*38 ± 0*.*20	125.07	0.31	636M
SAM ViT-L	0*.*39 ± 0*.*21	71.33	0.17	308M
SAM ViT-B	0*.*40 ± 0*.*22	37.54	0.09	86M
MedSAM	0*.*33 ± 0*.*23	30.43	0.07	86M

*DSC* Dice similarity coefficient, *MedSAM* medical segment anything model, *SAM* segment anything model, *ViT* vision transformer

Data are presented as mean ± standard deviation.

### 3.2 Impact of fine-tuning and composite loss function

**Fig. 5:**
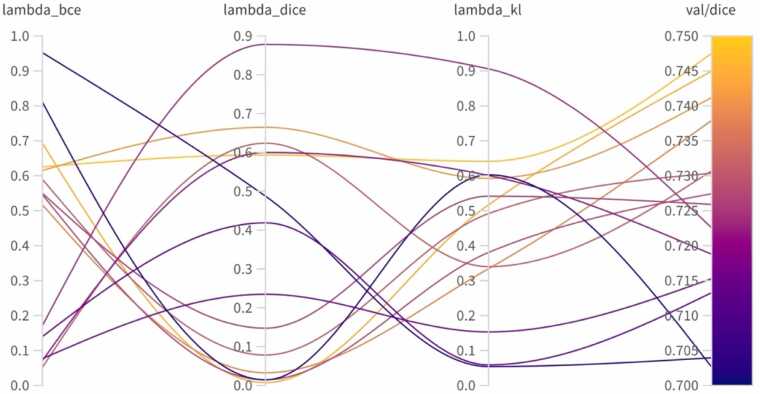
Loss weight optimization for segmentation performance. Parallel coordinates plot illustrating the hyperparameter optimization of the composite loss function. Each line represents a different combination of loss weights and, with color indicating the validation DSC. The optimal configuration was identified as: λ_BCE_ = 0.62, λ_Dice_ = 0.60, λ_KL_ = 0.64. *DSC* Dice similarity coefficient

**Fig. 6:**
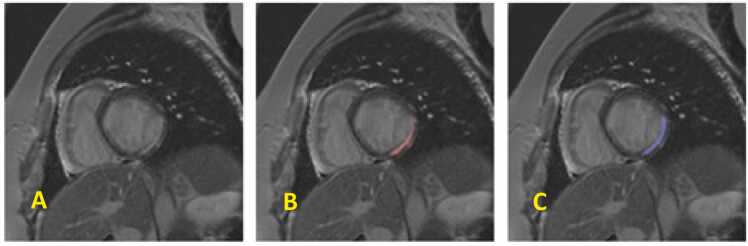
Segmentation outcomes after composite loss fine-tuning. Segmentation results after fine-tuning using the proposed composite loss function, shown on the same LGE-CMR slice as in Fig. 4. (A) Original image. (B) Ground truth scar annotation (red overlay). (C) Segmentation prediction after fine-tuning (blue overlay). Compared to the baseline (Fig. 4), fine-tuning leads to improved alignment with the ground truth in terms of boundary accuracy and scar completeness. *LGE* late gadolinium enhancement, *CMR* cardiovascular magnetic resonance

We evaluated the effect of Gaussian smoothing applied to soft labels through a grid search over the parameter *σ*. The best validation performance was consistently achieved with *σ* = 2, indicating that moderate smoothing effectively mitigates boundary noise and enhances model generalization. This setting was used in all subsequent experiments **(**Table 3**)**. Optimization of the composite loss function identified weighting factors ($\lambda_{\text{BCE}}$ = 0.62, $\lambda_{\text{Dice}}$ = 0.60, $\lambda_{\text{KL}}$ = 0.64) that achieved the highest DSC on the validation set (Fig. 5). Fine-tuning the ViT-B model with these optimized parameters significantly improved segmentation accuracy, increasing the DSC from 0.40 ± 0.22 (zero-shot) to 0.67 ± 0.12, and resulting in clearer delineation of myocardial scar boundaries (Fig. 6).

**Table 3:** Validation Dice scores for different values of the Gaussian smoothing parameter *σ*.

*σ*	0.3	0.5	0.7	0.9	1.1	1.3	1.5	2.0
Dice score	0.6548	0.6583	0.6597	0.6628	0.6597	0.6567	0.6632	**0.6697**

Bold indicates the σ that achieved the highest Dice score on the validation set.

**Table 4:** Segmentation performance across prompting strategies.

Prompt type	DSC score	Hausdorff distance
YOLO-based (automated)	0*.*60±0*.*33	10*.*73±14*.*22
Points only	0*.*69±0*.*12	9*.*31±10*.*05
Bounding box only	0*.*67±0*.*15	6*.*94±6*.*50
Bounding box + points	0*.*74±0*.*10	5*.*87±6*.*79

Data presented as mean ± SD

*DSC* Dice similarity coefficient, *SD* standard deviation, *YOLO* You Only Look Once

### 3.3 Interactive prompting improves segmentation accuracy

We assessed the impact of different prompting strategies on segmentation performance using the fine-tuned SAM model. The automated pipeline using YOLO-generated boxes achieved the lowest performance (DSC: 0*.*60 ± 0*.*33, HD: 10*.*73 ± 14*.*22), consistent with the findings from our previous MICCAI study [23], which highlighted the limitations of end-to-end prompting in heterogeneous clinical data. Manual interaction significantly improved accuracy. Point-based prompting yielded a DSC score of 0*.*69 ± 0*.*12, and bounding boxes alone reached 0*.*67 ± 0*.*15. The combined use of boxes and points provided the best performance (DSC: 0*.*74 ± 0*.*10, HD: 5*.*87 ± 6*.*79) (Table 4), indicating that spatially complementary prompts offer synergistic guidance. Patient-level scar mass error was 1.28 g (IQR 0.74–2.34), corresponding to 1.4% (IQR 0.81–2.47) of left ventricular myocardial mass. A representative visual comparison of points-only, bounding box only, and combined prompting strategies is shown in Supplementary Fig. S3. Segmentation performance did not differ significantly between infarct territories (one-way ANOVA, *p* = 0.26; Supplementary Table S2).

### 3.4 Repeatability and method agreement analysis

**Fig. 7:**
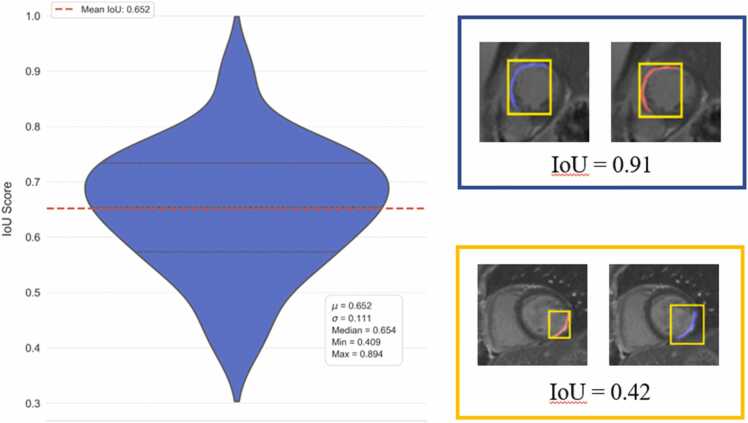
Inter-rater variability in bounding box overlap (IoU). Distribution of IoU scores between bounding boxes drawn by two raters. The red dashed line represents the mean IoU (0.65), illustrating the moderate variability in initial prompts used to guide the segmentation process. Inset examples show high overlap (IoU = 0.91, top) and low overlap (IoU = 0.42, bottom) between raters' bounding boxes. *IoU* intersection over union

**Fig. 8:**
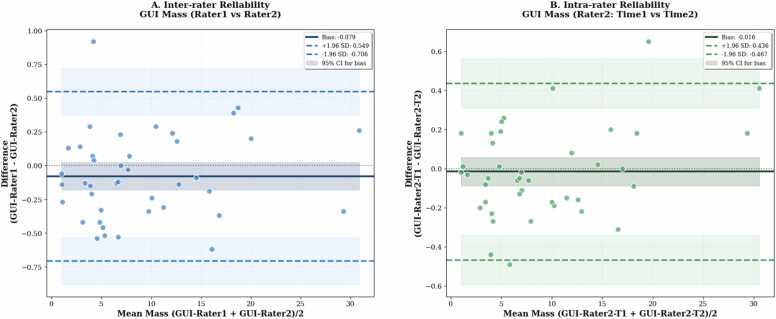
Repeatability of GUI-based scar mass quantification. Bland–Altman analysis of GUI-based myocardial scar mass quantification. (A) Inter-rater agreement (rater 1 vs rater 2). (B) Intra-rater agreement (rater 2, time 1 vs time 2). The GUI method demonstrates minimal bias and narrow 95% limits of agreement, indicating excellent repeatability. *GUI* graphical user interface, *SD* standard deviation, *CI* confidence interval

**Fig. 9:**
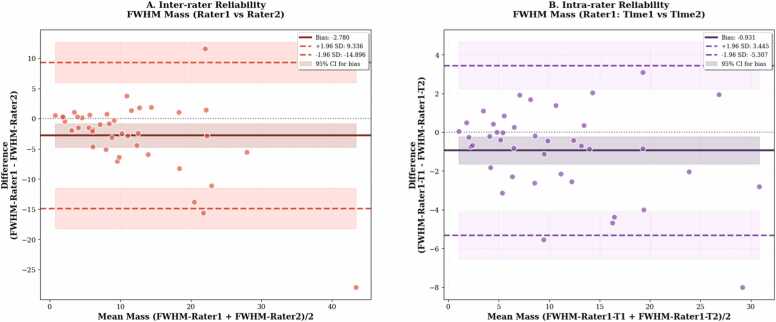
Repeatability of FWHM-based scar mass quantification. Bland–Altman analysis of FWHM-based scar mass quantification. (A) Inter-rater agreement (rater 1 vs rater 2). (B) Intra-rater agreement (rater 1, time 1 vs time 2). The FWHM method shows larger measurement variability and wider limits of agreement compared to the GUI-based approach. *FWHM* full-width at half-maximum method, *GUI* graphical user interface, *SD* standard deviation, *CI* confidence interval

**Fig. 10:**
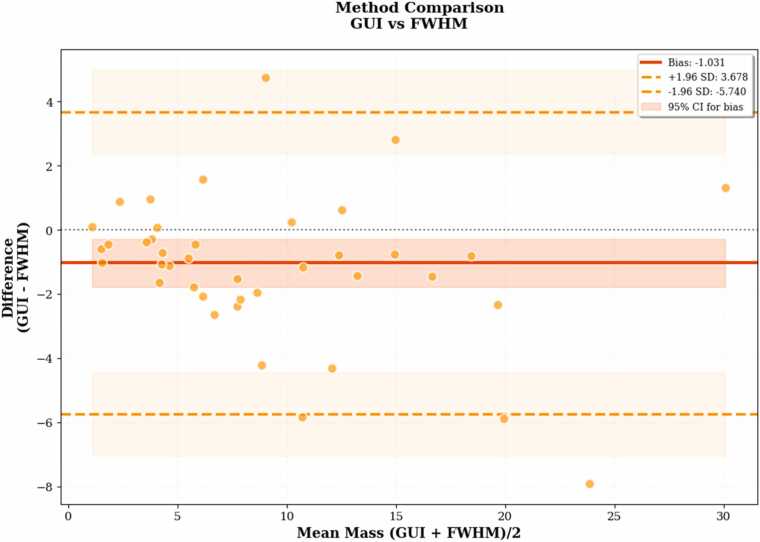
Comparison of GUI vs FWHM scar mass measurements. Bland–Altman plot comparing scar mass measurements between GUI- and FWHM-based methods. The FWHM method consistently underestimates scar mass relative to the GUI approach (mean bias = −1.031 g), despite strong correlation and concordance. *FWHM* full-width at half-maximum method, *GUI* graphical user interface, *SD* standard deviation, *CI* confidence interval

To evaluate the consistency of the proposed GUI-based segmentation method, we assessed both inter- and intra-rater reliability across 41 cases. The initial bounding boxes provided by different raters showed moderate variability (mean intersection over union (IoU) = 0.65, Fig. 7), indicating differences in the prompts used to initialize the segmentation for the same image. Inter-rater agreement between two independent experts demonstrated near-perfect consistency, with a mean bias of −0*.*079 g, SD of 0*.*320 g, ICC of 0.999, and CCC of 0.999 (Fig. 8). Intra-rater reliability showed similarly high repeatability (bias = −0*.*016 g, ICC = 0.999, CCC = 0.999; Fig. 8). Coefficients of variation (CV) were low in both comparisons (3.5% and 2.5%, respectively), indicating minimal measurement variability. In contrast, FWHM-based quantification exhibited greater variability: inter-rater comparisons yielded a bias of −2*.*780 g (SD = 6*.*182 g), with ICC = 0.737, CCC = 0.738, and CV = 53.6% (Fig. 9). Intra-rater agreement improved (bias = −0*.*931 g, SD = 2*.*232 g), with ICC = 0.952, CCC = 0.951, and CV = 21.1% (Fig. 9), though still less consistent than the GUI-based method. Method comparison between GUI- and FWHM-based scar mass quantification revealed strong concordance. For the same expert, ICC reached 0.936, CCC 0.935, and *r* = 0*.*945 (Fig. 10). However, agreement was lower when comparing across different raters (GUI: rater 1, FWHM: rater 2), with ICC = 0.728 and CCC = 0.736. The average analysis time using the GUI-based system was 65 ± 34 s per patient, including both automated inference and user interaction. In comparison, FWHM-based analysis using automated endocardial and epicardial contour detection (cvi42) with manual correction and exclusion zone placement required 368 ± 137 s (6.1 ± 2.3 min) per patient. Exclusion zones were applied in 98.1% of cases (51/52), with median excluded mass of 3.6 g (range: 0.0–27.8 g).

### 3.5 Generalization across imaging vendors and test set heterogeneity

To characterize the variability in imaging quality and signal characteristics within the test cohort, we computed seven quantitative metrics for each LGE-CMR slice: signal-to-noise ratio (SNR), contrast-to-noise ratio, which were measured between healthy myocardium and scar tissue, while image entropy, dynamic range, image intensity coefficient of variation, and Blind/Referenceless Image Spatial Quality Evaluator (BRISQUE) score [34] were calculated over entire slice, and number of the scar pixels. The test set demonstrated substantial heterogeneity: SNR ranged from 0.60 to 47.41 (mean 8.83 ± 5.97), BRISQUE score from 7.09 to 72.01 (mean 39.04 ± 13*.*54), and dynamic range from 82 to 255 (225.06 ± 41.73) (Table 5).

**Table 5:** Test set characteristics and summary of image quality and heterogeneity metrics.

Features	Min	Max	Mean±SD
Signal-to-noise ratio	0.60	47.41	8.83±5.97
Contrast-to-noise ratio	0.05	7.40	3.01±1*.*41
Image entropy	5.37	7.74	6.77±0.47
Dynamic range	82.0	255.0	225.06±41.73
Image intensity CoV (%)	10.09	118.83	62*.*43±30.88
BRISQUE score	7.09	72.01	39.04±13.54
Ground truth pixels	1	1516	209.95±202.83
DSC (per slice)	0.35	0.92	0.74±0.10

*BRISQUE* Blind/Referenceless Image Spatial Quality Evaluator, *CoV* coefficient of variation, *DSC* Dice similarity coefficient, *SD* standard deviation

Data are presented as mean ± standard deviation.

**Fig. 11:**
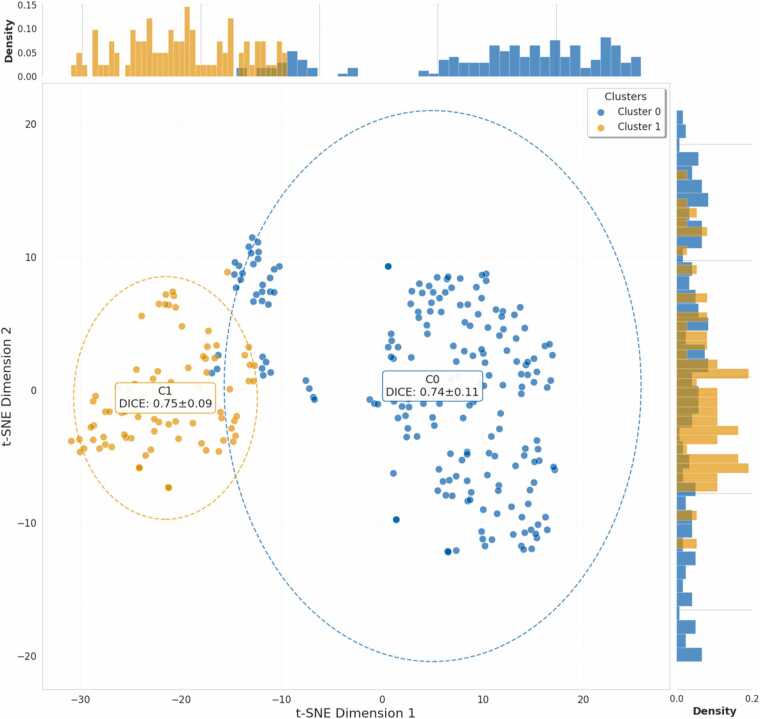
t-SNE clustering of test set heterogeneity with Dice performance. Visualization of test set heterogeneity and clustering results. t-SNE visualization of seven quantitative features, with k-means clustering (k = 2) identifying two groups (blue = Cluster 0, orange = Cluster 1). Marginal histograms show the distribution of each cluster along both t-SNE dimensions. Mean Dice scores (±SD) indicate comparable segmentation performance despite variability in image quality and acquisition characteristics, with no statistically significant difference between clusters (Mann–Whitney U test, *p* = 0.90). *tSNE t-distributed stochastic neighbor embedding*

These metrics confirm the inclusion of both low- and high-quality scans, reflecting substantial variability in image quality and heterogeneity across test set. To further explore this heterogeneity, the seven features were analyzed using K-means clustering, with the silhouette score indicating that the optimal number of clusters was K = 2. The clustering results were then visualized using t-SNE for dimensionality reduction (Fig. 11). Despite clear separation between the two clusters, which represent distinct image characteristic profiles based on the combined metrics, segmentation performance remained stable, with DSC of 0.74 ± 0.11 for cluster 0 and 0.75 ± 0.09 for cluster 1, indicating that the model maintained robust performance across images with diverse quality.

## 4 Discussion

This study represents an uncertainty-aware, interactive, deep learning framework for myocardial scar segmentation, achieving expert-level segmentation accuracy with excellent repeatability. Our approach addresses the key trade-off between accuracy and efficiency in CMR analysis through three innovations: (1) soft-label supervision with Kullback–Leibler divergence for boundary uncertainty, (2) prompt-resilient architecture maintaining performance despite variable user input, and (3) clinician-in-the-loop design that preserves interpretability while accelerating workflow.

Early deep learning approaches for LGE-CMR scar segmentation generally reported modest accuracy and limited generalizability. Semi-automated pipelines based on fully convolutional networks or U-Net architectures achieved DSC scores of only 0.57–0.71 when restricted to scar within a pre-segmented myocardium [19], [35]. Patch-based two-dimensional (2D)/three-dimensional (3D) convolutional neural networks (CNNs) showed higher DSC values (∼0.94), but these results were derived from a small single-center cohort (34 patients) and lacked validation under varied acquisition conditions [36]. 3D CNNs were subsequently explored to leverage volumetric context, but remained constrained by computational demands, sensitivity to variable slice coverage, and only moderate spatial overlap for scar delineation [37].

Even models incorporating synthetic data or generative adversarial network (GAN)-based augmentation improved performance only marginally. For instance, a cascaded cycle GAN pipeline achieved a mean per-subject DSC of 0.67 with a large spread (SD =0.29) and still required manual correction in many cases [38]. These limitations were underscored in a recent multi-center CMR segmentation challenge (MyoPS), where the average Dice for left ventricular scar was just 0.61 [39], illustrating that fully automated methods still struggle to produce consistent scar contours across heterogeneous data.

A central obstacle to scar segmentation is the presence of an ill-defined boundary between infarcted and healthy myocardium, caused by partial volume effects, low contrast differences, and imaging artifacts. It is also important to note that some of this boundary uncertainty reflects the recognized peri-infarct “grey zone,” a well-described transitional region in LGE-CMR that lacks a sharply defined boundary. Existing models are trained on homogeneous datasets and rely on single-step, fully automated predictions without user interaction or refinement. This makes them highly sensitive to noisy or out-of-distribution images [40]. When we benchmarked leading automated networks (nnU-Net, DeepLabv3+) on the same heterogeneous dataset, DSC scores dropped to 0.57–0.59 [23] and performance deteriorated on low-quality scans, highlighting their lack of robustness. Earlier non–learning-based approaches attempted to stabilize LGE analysis by transferring cine-derived LV contours, achieving sub-pixel agreement with expert borders (0.82 ± 0.19 pixels), but focusing on myocardial border definition rather than scar delineation itself [41]. Deep learning approaches have similarly incorporated additional anatomical context within fully automated scar segmentation pipelines, without providing a mechanism for incorporating expert input at inference [42].

In contrast, our human-in-the-loop framework couples uncertainty-aware training with real-time user guidance: initial prompts are automatically generated using YOLO, and clinicians can refine these prompts or add point-based inputs when needed. The model then refines the segmentation based on the final user inputs. This interactive paradigm not only yields a higher mean Dice (0.74) but also maintains performance across heterogeneous data: unlike static pipelines, it allows the user to modulate the segmentation to reflect subtle scar–myocardium interfaces and imaging nuances. Such a hybrid approach aligns with recent evidence from other domains (e.g., liver and orbital MRI) where expert-corrected AI segmentations significantly reduced inter-observer variability and segmentation time [43], suggesting that clinician-guided AI is important for safe and effective deployment in cardiac imaging. By incorporating expert feedback at the point of care, our system bridges the gap between fully automated algorithms and the variability of real-world clinical data.

### 4.1 Clinical implications

Scar quantification is a powerful predictor of adverse outcomes, yet it is underutilized in routine clinical practice due to the time required and poor reproducibility of existing methods. The intersection of repeatability and efficiency defines clinical viability in cardiac imaging. Our framework’s near-perfect inter- and intra-observer agreement (ICC > 0.99) eliminates the 30–50% variability that currently plagues scar quantification [11], [18], which may adversely impact risk stratification and downstream clinical decisions, such as regarding implantable cardiac defibrillator eligibility and revascularization. Our uncertainty-aware approach maintains consistency where expert consensus fails (DSC = 0.52) in anatomically complex regions dominated by partial volume effects [44]. Critically, this precision comes without a temporal penalty. While fully manual LGE analysis has been reported to require up to 20–30 min per patient [45], modern FWHM workflows using automated contour detection still required 6.1 min in our study due to the need for manual contour correction and exclusion zone placement. The proposed GUI reduced analysis time to approximately 1 min while preserving interactive control, replacing detailed contour editing with simple prompt-based guidance that integrates clinical judgment directly into the inference process. By addressing these limitations, our framework may improve the feasibility of scar quantification in routine clinical practice and facilitate future research into its diagnostic and prognostic roles in guiding patient management.

### 4.2 Technical innovations

Our system embodies three key technical contributions, each translating directly to practical clinical benefits: soft-label supervision with uncertainty awareness, interactive inference with robustness to prompt variability, and lightweight deployment with a clinician-centered GUI. Instead of relying on binary masks, our training utilizes smoothed labels combined with a loss that includes KL divergence. This approach explicitly models uncertainties near the interface between scarred and healthy myocardium, where boundaries are often unclear due to partial volume effects, diffuse fibrosis, or imaging artifacts [44]. Soft-label training has been shown to improve segmentation calibration and sensitivity to subtle tissue changes in other imaging domains [46], [47].

Our framework enables prompt-driven interaction that is resilient to variability in input type and location [48], [49]; despite substantial differences in bounding boxes placed by independent raters (mean IoU 0.65), scar mass agreement remained near-perfect (CCC 0.999). Unlike static models such as nnU-Net [50], which lack the flexibility to adapt to user guidance, our system produces high-quality outputs guided by coarse user input, without requiring iterative correction or retraining. This is especially impactful, where imaging quality, infarct morphology, and acquisition protocols vary across cases. By aligning predictions with user guidance in real time, the system maintains performance across difficult scenarios, while preserving expert control.

Inter-observer segmentation differences often reflect subjective variation in prompts—for example, drawing bounding boxes tightly or loosely around the region of interest. By training the model to handle a range of plausible prompts, we encode this variability, promoting robustness and clinical usability.

For clinical AI systems to translate beyond research settings, they must operate within real-world computational and workflow constraints. Inference completes in under a few seconds per volume, and segmentation adjustments such as shrink or expand are applied directly through the graphical interface without triggering secondary pipelines. Automated region proposals, initialized by a lightweight object detector, allow users to begin with a suggested region of interest, which can then be refined using intuitive point or box prompts; similar prompt-based interfaces have been shown to dramatically reduce annotation time while preserving diagnostic quality [43], [51].

## 5 Limitations

Several limitations should be acknowledged. First, although our system was evaluated across multiple centers and vendors, external validation on larger external datasets is necessary to confirm generalizability before clinical deployment. Second, the current study focused exclusively on post-ST-segment elevation MI patients with chronic infarcts in whom microvascular obstruction has typically resolved; applicability to acute infarction and non-ischemic cardiomyopathies is untested. Third, the initial bounding box detection using our YOLO-based pipeline demonstrated moderate performance and may require user correction in complex cases, suggesting a potential failure point that merits further refinement. From a technical standpoint, the model currently operates in a 2D slice-wise fashion, which may limit its ability to capture through-plane anatomical continuity. Future work should explore 3D or hybrid 2.5D architectures, as well as longitudinal constraints to improve spatial coherence across slices. Fourth, as with all supervised medical imaging models, performance is bounded by ground truth quality. We selected FWHM-based labeling as Flett et al. [12] demonstrated it to be the most reproducible method for ischemic scar quantification; nevertheless, residual reference variability imposes a ceiling on achievable model performance, a limitation shared by all fine-structure medical image segmentation tasks [52]. Additionally, although the system is computationally lightweight and compatible with standard systems, seamless integration into hospital radiology analysis workflows and navigation of regulatory pathways will be critical for real-world deployment.

## 6 Future work

Future research should pursue several directions: (1) prospective validation in real clinical workflows, with measurement of impact on downstream decisions such as device eligibility or revascularization planning; (2) extension of the framework to broader cardiac pathologies, including non-ischemic fibrosis and diffuse myocardial disease, as well as characterization of the peri-infarct gray zone; (3) implementation of uncertainty quantification and visualization to highlight regions of low confidence, enhancing clinician trust; and (4) development of an active learning loop, whereby user corrections are systematically captured and used to iteratively improve the model, ensuring continued adaptation to evolving imaging protocols, populations, and clinical expectations.

## 7 Conclusions

This study introduces an interactive deep learning framework for myocardial scar segmentation from LGE-CMR, combining cutting-edge algorithms and clinical usability. By integrating uncertainty-aware training with clinician-guided prompts, the system offers both fast, consistent, and interpretable scar quantification across heterogeneous data. Its lightweight design and real-time human-in-the-loop architecture position it as a practical solution for standardizing CMR interpretation across diverse imaging environments.

## Funding

A.M. is funded by a 10.13039/501100000738University of Leicester Future 100 Studentship. M.M.G. is supported by the Pioneer Centre for AI (DNRF grant P1). J.R.A. is supported by an 10.13039/100006662NIHR EME Fellowship (170057). G.P.M. was supported by a NIHR Research Professorship (RP-2017-08-ST2-007).

## Author contributions

**J. Ranjit Arnold:** Writing – review & editing, Validation, Supervision, Resources, Methodology, Funding acquisition, Conceptualization. **Aida Moafi:** Writing – review & editing, Writing – original draft, Visualization, Validation, Software, Methodology, Investigation, Formal analysis, Data curation, Conceptualization. **Gerry P. McCann:** Writing – review & editing, Supervision, Resources. **Nilesh J. Samani:** Writing – review & editing, Resources. **Mostafa Mehdipour Ghazi:** Writing – review & editing, Supervision, Methodology, Conceptualization. **Simran Shergill:** Writing – review & editing, Validation. **Danial Moafi:** Writing – review & editing, Validation, Software, Methodology, Formal analysis, Conceptualization. **David Adlam:** Writing – review & editing, Resources. **Evgeny M. Mirkes:** Writing – review & editing, Supervision, Data curation.

## Declaration of competing interests

G.P.M. is an editorial board member for JCMR, but was not involved in the peer review of this article and had no access to information regarding its peer review. Full responsibility for the editorial process for this article was delegated to another journal editor. The remaining authors have no competing interests to declare.

## Data Availability

The source code and pretrained weights for the proposed segmentation framework will be made publicly available at: https://github.com/Danialmoa/SAM-MedUI. Due to institutional and ethical constraints, the patient imaging data used in this study cannot be publicly released.

## References

[1] Lindstrom M., DeCleene N., Dorsey H., Fuster V., Johnson C.O., LeGrand K.E. (2022). Global burden of cardiovascular diseases and risks collaboration, 1990-2021. J Am Coll Cardiol.

[2] Timmis A., Aboyans V., Vardas P., Townsend N., Torbica A., Kavousi M. (2024). European Society of Cardiology: the 2023 atlas of cardiovascular disease statistics. Eur Heart J.

[3] Hall M., Smith L., Wu J., Hayward C., Batty J.A., Lambert P.C. (2024). Health outcomes after myocardial infarction: a population study of 56 million people in England. PLos Med.

[4] Stone G.W., Selker H.P., Thiele H., Patel M.R., Udelson J.E., Ohman E.M. (2016). Relationship between infarct size and outcomes following primary PCI: patient-level analysis from 10 randomized trials. J Am Coll Cardiol.

[5] Disertori M., Rigoni M., Pace N., Casolo G., Mase M., Gonzini L. (2016). Myocardial fibrosis assessment by LGE is a powerful predictor of ventricular tachyarrhythmias in ischemic and nonischemic LV dysfunction: a meta-analysis. JACC Cardiovasc Imaging.

[6] Jablonowski R., Chaudhry U., Van Der Pals J., Engblom H., Arheden H., Heiberg E. (2017). Cardiovascular magnetic resonance to predict appropriate implantable cardioverter defibrillator therapy in ischemic and nonischemic cardiomyopathy patients using late gadolinium enhancement border zone: comparison of four analysis methods. Circ Cardiovasc Imaging.

[7] Acosta J., Fernndez-Armenta J., Borrs R., Anguera I., Bisbal F., Mart-Almor J. (2018). Scar characterization to predict life-threatening arrhythmic events and sudden cardiac death in patients with cardiac resynchronization therapy: the GAUDI-CRT study. JACC Cardiovasc Imaging.

[8] Kancharla K., Weissman G., Elagha A.A., Kancherla K., Samineni S., Hill P.C. (2016). Scar quantification by cardiovascular magnetic resonance as an independent predictor of long-term survival in patients with ischemic heart failure treated by coronary artery bypass graft surgery. J Cardiovasc Magn Reson.

[9] Helali J., Ramesh K., Brown J., Preciado-Ruiz C., Nguyen T., Silva L.T. (2025). Late gadolinium enhancement on cardiac MRI: a systematic review and meta-analysis of prognosis across cardiomyopathies. Int J Cardiol.

[10] Meier C., Eisenbltter M., Gielen S. (2024). Myocardial late gadolinium enhancement (LGE) in cardiac magnetic resonance imaging (CMR)—an important risk marker for cardiac disease. J Cardiovasc Dev Dis.

[11] Zhang L., Huttin O., Marie P.-Y., Felblinger J., Beaumont M., Chillou C.D.E. (2016). Myocardial infarct sizing by late gadolinium-enhanced MRI: comparison of manual, full-width at half-maximum, and n-standard deviation methods. J Magn Reson Imaging.

[12] Flett A.S., Hasleton J., Cook C., Hausenloy D., Quarta G., Ariti C. (2011). Evaluation of techniques for the quantification of myocardial scar of differing etiology using cardiac magnetic resonance. JACC Cardiovasc Imaging.

[13] Khan J.N., Nazir S.A., Horsfield M.A., Singh A., Kanagala P., Greenwood J.P. (2015). Comparison of semi-automated methods to quantify infarct size and area at risk by cardiovascular magnetic resonance imaging at 1.5 T and 3.0 T field strengths. BMC Res Notes.

[14] Heiberg E., Engblom H., Carlsson M., Erlinge D., Atar D., Aletras A.H. (2022). Infarct quantification with cardiovascular magnetic resonance using standard deviation from remote is unreliable: validation in multi-centre multi-vendor data. J Cardiovasc Magn Reson.

[15] Jathanna N., Podlasek A., Sokol A., Auer D., Chen X., Jamil-Copley S. (2021). Diagnostic utility of artificial intelligence for left ventricular scar identification using cardiac magnetic resonance imaging-a systematic review. Cardiovasc Digit Health J.

[16] Moafi A., Dattani A., Samani N., Mirkes E.M., McCann G., Arnold J.R. (2024). 190 Quantification of myocardial infarction by cardiovascular magnetic resonance late gadolinium enhancement imaging: comparison of magnitude-based versus phase-sensitive inversion recovery. Heart.

[17] Burger J.C., Hopman L.H.G.A., Campos F.O., Allaart C.P., Postema P.G., Kemme M.J.B. (2025). Optimizing ventricular scar characterization in late-gadolinium enhancement cardiac MRI: impact of thresholding techniques in magnitude and phase-sensitive reconstructed images. Heart Rhythm.

[18] Karim R., Bhagirath P., Claus P., Housden R.J., Chen Z., Karimaghaloo Z. (2016). Evaluation of state-of-the-art segmentation algorithms for left ventricle infarct from late gadolinium enhancement MR images. Med Image Anal.

[19] Moccia S., Banali R., Martini C., Muscogiuri G., Pontone G., Pepi M. (2019). Development and testing of a deep learning-based strategy for scar segmentation on CMR-LGE images. Magn Reson Mater Phys Biol Med.

[20] Zabihollahy F., White J.A., Ukwatta E. (2019). Convolutional neural network-based approach for segmentation of left ventricle myocardial scar from 3D late gadolinium enhancement MR images. Med Phys.

[21] Tavakoli N., Rahsepar A.A., Benefield B.C., Shen D., López-Tapia S., Schiffers F. (2025). ScarNet: a novel foundation model for automated myocardial scar quantification from late gadolinium-enhancement images. J Cardiovasc Magn Reson.

[22] Lalande A., Chen Z., Pommier T., Decourselle T., Qayyum A., Salomon M. (2022). Deep learning methods for automatic evaluation of delayed enhancement-MRI. The results of the EMIDEC challenge. Med Image Anal.

[23] Moafi A, Moafi D, Mirkes EM, McCann GP, Alatrany AS, Arnold JR, et al, editors. Robust deep learning for myocardial scar segmentation in cardiac MRI with noisy labels. International Conference on Medical Image Computing and Computer-Assisted Intervention; 2025. Springer.

[24] Vanezis A.P., Arnold J.R., Rodrigo G., Lai F.Y., Debiec R., Nazir S. (2018). Daily remote ischaemic conditioning following acute myocardial infarction: a randomised controlled trial. Heart.

[25] McCann G.P., Khan J.N., Greenwood J.P., Nazir S., Dalby M., Curzen N. (2015). Complete versus lesion-only primary PCI: the randomized cardiovascular MR CvLPRIT substudy. J Am Coll Cardiol.

[26] Persu A., Lopez-Sublet M., Al-Hussaini A., Pappaccogli M., Radhouani I., Van der Niepen P. (2022). Prevalence and disease spectrum of extracoronary arterial abnormalities in spontaneous coronary artery dissection. JAMA Cardiol.

[27] Khan J.N., Razvi N., Nazir S.A., Singh A., Masca N.G., Gershlick A.H. (2014). Prevalence and extent of infarct and microvascular obstruction following different reperfusion therapies in ST-elevation myocardial infarction. J Cardiovasc Magn Reson.

[28] Prez-Garca F., Sparks R., Ourselin S. (2021). TorchIO: a Python library for efficient loading, preprocessing, augmentation and patch-based sampling of medical images in deep learning. Comput Methods Prog Biomed.

[29] Zuiderveld K., Heckbert P.S. (1994). Graphics Gems, vol. IV.

[30] Kirillov A, Mintun E, Ravi N, Mao H, Rolland C, Gustafson L, et al, editors. Segment anything. Proceedings of the IEEE/CVF International Conference on Computer Vision; 2023.

[31] Ma J., He Y., Li F., Han L., You C., Wang B. (2024). Segment anything in medical images. Nat Commun.

[32] Biewald L. Experiment tracking with weights and biases. Software available at https://www.wandb.com/. 2020.

[33] Bonett D.G. (2002). Sample size requirements for estimating intraclass correlations with desired precision. Stat Med.

[34] Van Eeden Risager K, Gholamalizadeh T, Mehdipour Ghazi M, editors. Non-reference quality assessment for medical imaging: application to synthetic brain MRIs. MICCAI Workshop on Deep Generative Models; 2024. Springer.

[35] Fahmy A.S., Rausch J., Neisius U., Chan R.H., Maron M.S., Appelbaum E. (2018). Automated cardiac MR scar quantification in hypertrophic cardiomyopathy using deep convolutional neural networks. JACC Cardiovasc Imaging.

[36] Zabihollahy F., Rajan S., Ukwatta E. (2020). Machine learning-based segmentation of left ventricular myocardial fibrosis from magnetic resonance imaging. Curr Cardiol Rep.

[37] Fahmy A.S., Neisius U., Chan R.H., Rowin E.J., Manning W.J., Maron M.S. (2020). Three-dimensional deep convolutional neural networks for automated myocardial scar quantification in hypertrophic cardiomyopathy: a multicenter multivendor study. Radiology.

[38] Lustermans D.R., Amirrajab S., Veta M., Breeuwer M., Scannell C.M. (2022). Optimized automated cardiac MR scar quantification with GAN-based data augmentation. Comput Methods Prog Biomed.

[39] Li L., Wu F., Wang S., Luo X., Martn-Isla C., Zhai S. (2023). MyoPS: a benchmark of myocardial pathology segmentation combining three-sequence cardiac magnetic resonance images. Med Image Anal.

[40] Reinke A, Tizabi MD, Sudre CH, Eisenmann M, Rdsch T, Baumgartner M, et al. Common limitations of image processing metrics: a picture story. arXiv preprint arXiv:2104.05642; 2021.

[41] Tao Q., Piers S.R., Lamb H.J., van der Geest R.J. (2015). Automated left ventricle segmentation in late gadolinium‐enhanced MRI for objective myocardial scar assessment. J Magn Reson Imaging.

[42] Fahmy A.S., Rowin E.J., Chan R.H., Manning W.J., Maron M.S., Nezafat R. (2021). Improved quantification of myocardium scar in late gadolinium enhancement images: deep learning based image fusion approach. J Magn Reson Imaging.

[43] Chlebus G., Meine H., Thoduka S., Abolmaali N., Van Ginneken B., Hahn H.K. (2019). Reducing inter-observer variability and interaction time of MR liver volumetry by combining automatic CNN-based liver segmentation and manual corrections. PLoS One.

[44] Zhuang X. (2018). Multivariate mixture model for myocardial segmentation combining multi-source images. IEEE Trans Pattern Anal Mach Intell.

[45] Jani V.P., Ostovaneh M., Chamera E., Kato Y., Lima J.A.C., Ambale-Venkatesh B. (2024). Deep learning for automatic volumetric segmentation of left ventricular myocardium and ischaemic scar from multi-slice late gadolinium enhancement cardiovascular magnetic resonance. Eur Heart J Cardiovasc Imaging.

[46] Gros C., Lemay A., Cohen-Adad J. (2021). SoftSeg: advantages of soft versus binary training for image segmentation. Med Image Anal.

[47] Islam M., Glocker B., Feragen A., Sommer S., Schnabel J., Nielsen M. (2021). Information Processing in Medical Imaging.

[48] Cheng D, Qin Z, Jiang Z, Zhang S, Lao Q, Li K. SAM on medical images: a comprehensive study on three prompt modes. arXiv preprint arXiv:2305.00035. 2023.

[49] Ali M., Wu T., Hu H., Luo Q., Xu D., Zheng W. (2025). A review of the segment anything model (SAM) for medical image analysis: accomplishments and perspectives. Comput Med Imaging Graph.

[50] Isensee F., Jaeger P.F., Kohl S.A.A., Petersen J., Maier-Hein K.H. (2021). nnU-Net: a self-configuring method for deep learning-based biomedical image segmentation. Nat Methods.

[51] Chang Y.J., Cho J., Shon B., Choi K.Y., Jeong S., Ryu J.Y. (2025). A novel clinical investigation using deep learning and human-in-the-loop approach in orbital volume measurement. J CranioMaxillofac Surg.

[52] Maier-Hein L., Eisenmann M., Reinke A., Onogur S., Stankovic M., Scholz P. (2018). Why rankings of biomedical image analysis competitions should be interpreted with care. Nat Commun.

