# Supplementary material for: Interactive deep learning for myocardial scar segmentation using cardiovascular magnetic resonance
Source: J Cardiovasc Magn Reson. 2026 Mar 20;28(1):102720. doi: 10.1016/j.jocmr.2026.102720 (PMC13241718; doi:10.1016/j.jocmr.2026.102720)
Supplement: Supplementary file 2 — Supplementary material [file mmc1.docx]

**Supplementary Table S1.** **Summary of image acquisition parameters across cohorts**

| **Study** | **Manufacturer** | **Scanner**  **models** | **Field**  **Strength (Tesla)** | **Slice**  **thickness (mm)** | **Slice gap**  **(mm)** | **Image resolution** | **Pixel spacing (mm)** |
| --- | --- | --- | --- | --- | --- | --- | --- |
| SCAD | Siemens | Skyra | 3 | 8, 10 | 10 | 224×256, 176×256, 256×192, 192×256, 224×224 | 1.52×1.52, 1.37×1.37, 1.96×1.96, 1.25×1.25, 1.17×1.17, 1.48×1.48, 1.45×1.45 |
| CvLPRIT | Siemens, Philips | Avanto, Aera,  Intera | 1.5 | 8, 10 | 10 | 256×224, 256×208, 256×200, 256×256 | 1.56×1.56, 1.52×1.52, 1.48×1.48, 1.37×1.37, 1.25×1.25, 1.17×1.17, 1.33×1.33, 1.45×1.45, 1.41×1.41 |
| DREAM | Siemens | Avanto, Skyra | 1.5&3 | 8, 10 | 10 | 256×232, 224×168, 256×192, 256×216 | 1.66×1.66, 1.45×1.45, 1.52×1.52, 1.48×1.48, 1.37×1.37, 1.29×1.29, 1.79×1.79, 1.72×1.72 |
| AMI | Siemens | Avanto | 1.5 | 6 | 10 | 256×224, 256×208, 256×200 | 1.56×1.56, 1.52×1.52, 1.48×1.48, 1.64×1.64, 1.33×1.33, 1.72×1.72 |

**Abbreviations:** AMI= Acute Myocardial Infarction; CvLPRIT = Complete versus Lesion-only Primary Percutaneous Coronary Intervention trial; DREAM = Daily Remote Conditioning in Acute Myocardial Infarction trial; SCAD = Spontaneous Coronary Artery Dissection registry.

**Supplementary Table S2:** Segmentation performance by infarct territory

| **Territory** | **N** | **Dice (mean ± SD)** |
| --- | --- | --- |
| Anterior (LAD) | 19 | 0.75 ± 0.04 |
| Inferior (RCA) | 15 | 0.75 ± 0.06 |
| Lateral (LCx) | 7 | 0.73 ± 0.04 |
| Mixed* | 11 | 0.72 ± 0.08 |

*Patients with scar involving multiple coronary territories. No significant difference between territories (one-way ANOVA, p=0.26).

**Abbreviations:** LAD = left anterior descending; LCx = left circumflex; RCA = right coronary artery.


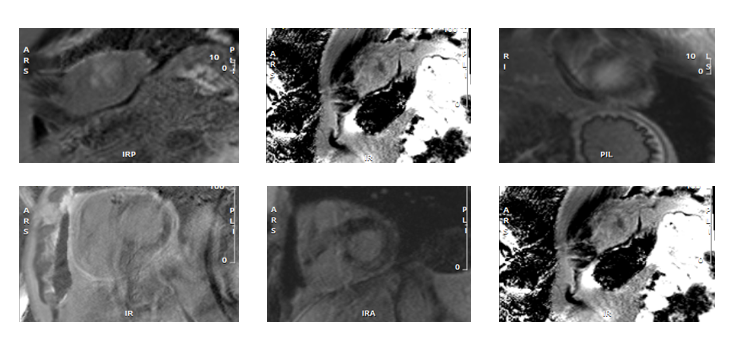
**Supplementary Figure S1.** **Cases excluded due to insufficient image quality**

Representative cases excluded due to insufficient image quality for ground truth annotation. Short-axis late gadolinium enhancement images demonstrating motion artefact, poor contrast-to-noise ratio, or insufficient signal intensity such that scar boundaries could not be consistently identified by expert observers.


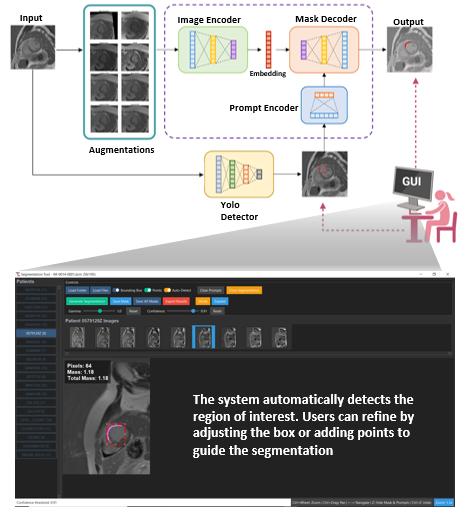
**Supplementary Figure S2.** **Overview of interactive segmentation GUI and workflow**

Overview of the interactive segmentation framework and graphical user interface. (Top) Model architecture comprising image encoder, prompt encoder, and mask decoder. A YOLO detector automatically generates initial bounding box prompts. (Bottom) The graphical user interface displaying a representative case. The system automatically detects the region of interest; users can refine by adjusting the bounding box or adding foreground points to guide segmentation. Real-time scar quantification is displayed (pixels, mass, total mass). See Supplementary Video S1 for demonstration.

**Supplementary Figure S3.** **Comparison of prompting strategies for scar segmentation**

Example comparison of points-only, bounding-box-only, and combined bounding-box plus points prompting for myocardial scar segmentation. The yellow dashed box indicates the zoomed region shown in the lower panels.

**
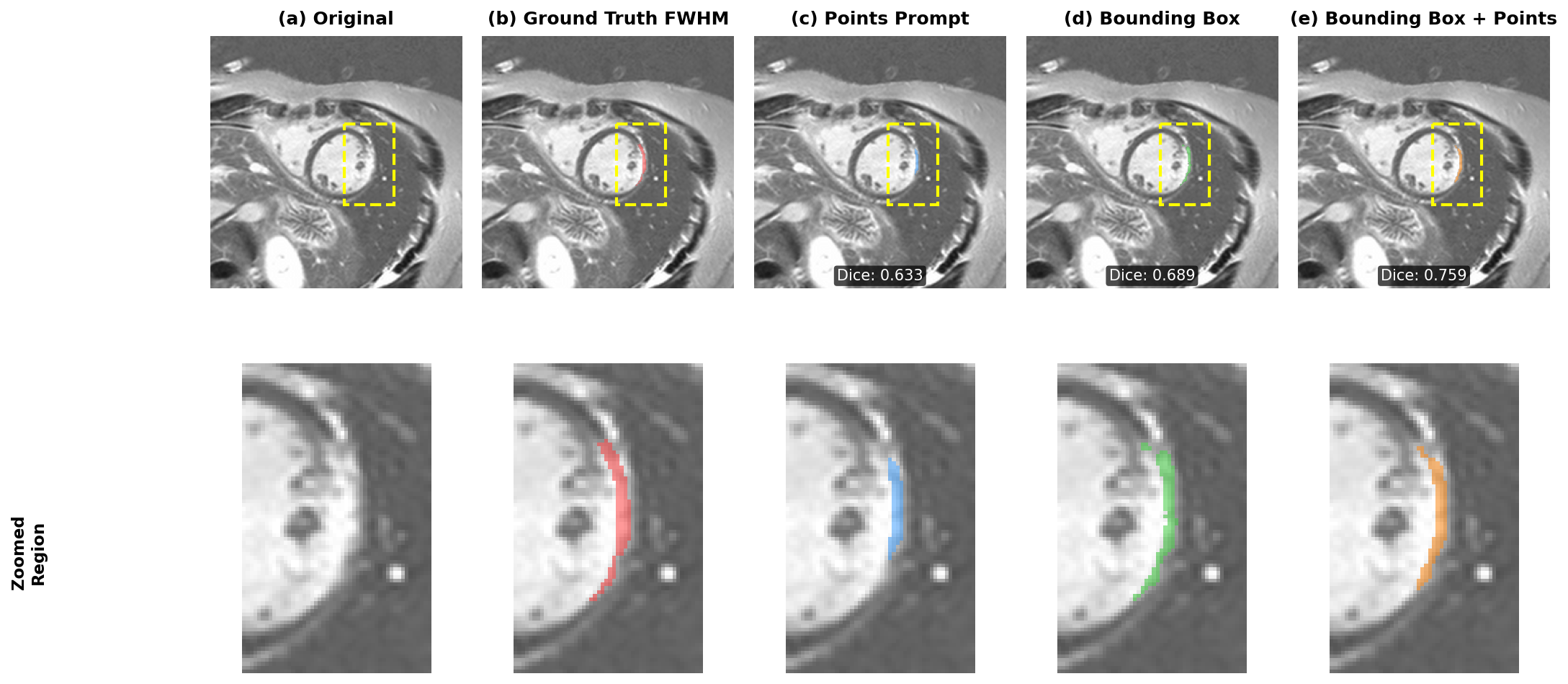
**

**Supplementary Figure S4.** **Representative test-set case with low Dice similarity**


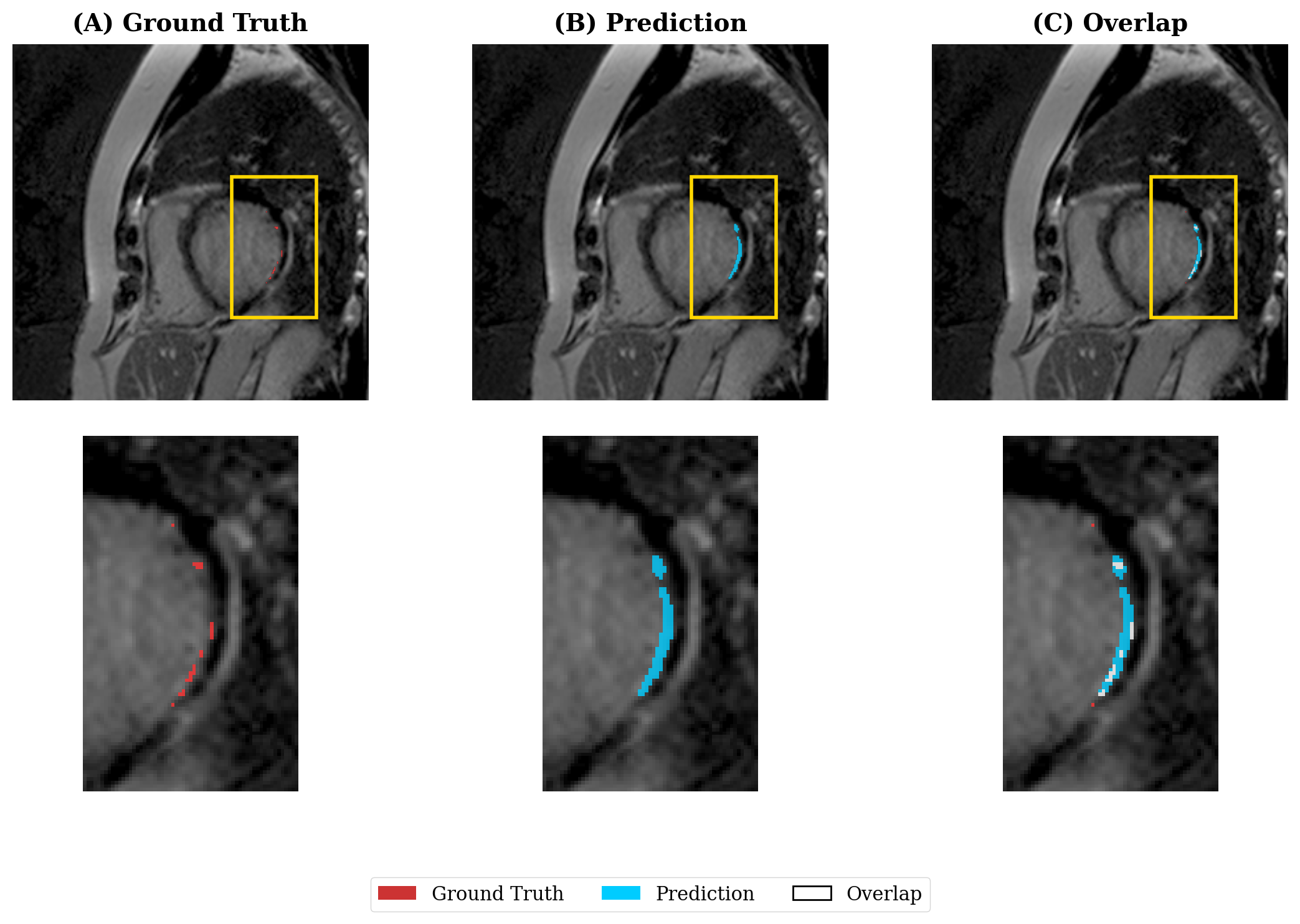


Representative slice demonstrating low Dice similarity coefficient (DSC = 0.35). (A) Ground truth scar annotation (red). (B) Model prediction (cyan). (C) Overlay showing both segmentations identifying enhancement in the same anatomical region but differing in extent at the scar-myocardium boundary. Lower panels show magnified views of the region of interest.
